# The landscape of nutri-informatics: a review of current resources and challenges for integrative nutrition research

**DOI:** 10.1093/database/baab003

**Published:** 2021-01-25

**Authors:** Lauren Chan, Nicole Vasilevsky, Anne Thessen, Julie McMurry, Melissa Haendel

**Affiliations:** College of Public Health and Human Sciences, Oregon State University, 101 Milam Hall, Corvallis, OR 97331, USA; Oregon Clinical and Translational Research Institute, Department of Medical Informatics and Clinical Epidemiology, Oregon Health and Science University, 3181 SW Sam Jackson Park Rd SN4N, Portland, OR 97239, USA; Environmental and Molecular Toxicology Department, Oregon State University, 1007 Ag & Life Sciences Building, Corvallis, OR 97331, USA; College of Public Health and Human Sciences, Oregon State University, 101 Milam Hall, Corvallis, OR 97331, USA; Oregon Clinical and Translational Research Institute, Department of Medical Informatics and Clinical Epidemiology, Oregon Health and Science University, 3181 SW Sam Jackson Park Rd SN4N, Portland, OR 97239, USA; Environmental and Molecular Toxicology Department, Oregon State University, 1007 Ag & Life Sciences Building, Corvallis, OR 97331, USA

## Abstract

Informatics has become an essential component of research in the past few decades, capitalizing on the efficiency and power of computation to improve the knowledge gained from increasing quantities and types of data. While other fields of research such as genomics are well represented in informatics resources, nutrition remains underrepresented. Nutrition is one of the most integral components of human life, and it impacts individuals far beyond just nutrient provisions. For example, nutrition plays a role in cultural practices, interpersonal relationships and body image. Despite this, integrated computational investigations have been limited due to challenges within nutrition informatics (nutri-informatics) and nutrition data. The purpose of this review is to describe the landscape of nutri-informatics resources available for use in computational nutrition research and clinical utilization. In particular, we will focus on the application of biomedical ontologies and their potential to improve the standardization and interoperability of nutrition terminologies and relationships between nutrition and other biomedical disciplines such as disease and phenomics. Additionally, we will highlight challenges currently faced by the nutri-informatics community including experimental design, data aggregation and the roles scientific journals and primary nutrition researchers play in facilitating data reuse and successful computational research. Finally, we will conclude with a call to action to create and follow community standards regarding standardization of language, documentation specifications and requirements for data reuse. With the continued movement toward community standards of this kind, the entire nutrition research community can transition toward greater usage of Findability, Accessibility, Interoperability and Reusability principles and in turn more transparent science.

## The emergence of nutri-informatics

The term ‘nutri-informatics’ describes approaches to understand the interactions between an organism and its nutritional environment via bioinformatics-based integration of nutrition study data sets (1). Nutri-informatics aims to computationally integrate and analyze nutrition study data sets in order to disentangle the interactions between an organism and its nutritional environment. Fueled by an interest in how food, nutrients and nutrition sociology impact health, and a recent push toward ‘big data’, nutri-informatics is essential to incorporating nutrition into computational biomedical sciences.

Nutri-informatics suffers from a lack of standardization with a wide array of groups working on similar projects with no community-wide development principles to ensure interoperability and cohesion between nutri-informatics and other biomedical resources. While a large number of resources for nutri-informatics are available, much of nutrition is underrepresented. This may be due to how expansive and heterogeneous nutrition is as a field, increasing the difficulty of data modeling. Approaches to formalize nutrition research language and connect standardized terminologies across biomedical fields have been initiated through the use of biomedical ontologies and computational nutrition data resources. While a variety of nutrition-related ontologies have been initiated, they are still in development and require further attention from nutrition researchers and biomedical ontologists.

Should nutrition data continue to be produced with no standardization of language, documentation specifications or requirements for data reuse, nutri-informatics investigations will continue to struggle with incompatible data. In an effort to support nutri-informatics, the community must encourage standards for nutrition data production, reuse and publication. Academic journals as well as members from nutrition research and biomedical ontology communities should promote standardization of language and data interoperability.

## Nutrition research encompasses a broad swath of human biology

While nutrition and diet are arguably some of the most vital aspects of a healthy life, the study of nutrition as a science is relatively new. Modern-day nutrition research began less than 100 years ago with the first vitamin isolation in 1926 (2) but has grown into a vast discipline. From a biological standpoint, nutrition is essential to all living organisms. Life, functions and reproduction of humans and other organisms are supported by essential nutrients such as water, macronutrients, vitamins and minerals obtained from food and drink. Thus, nutrition research has focused on understanding what nutrients are essential (3–5), what foods contain those nutrients (6, 7), what biological functions a nutrient may participate in (8, 9), how food processing impacts nutrient content (10–12) and evaluation of ideal nutrient needs for individuals with specific health conditions (13–15).

Evidence-based nutrition research has informed clinical and public health practices, such as adding folic acid to grain products due to the association between inadequate folate consumption by pregnant mothers and neural tube defects in the offspring. Clearly defining individual and population nutrition recommendations has been a consistent focus for disease prevention and management, and health optimization (16, 17). However, recent advances in understanding nutrient–nutrient interactions (18, 19), food–drug interactions (20), molecular processes and the impact of the microbiome (21, 22) make nutrition far more complex than initially thought.

**Figure 1.**
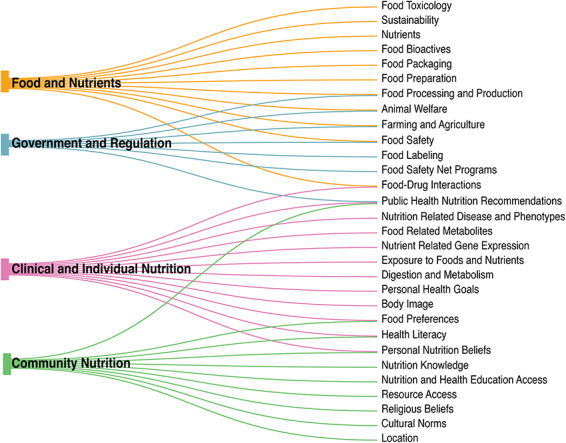
The nutri-informatics landscape. Nutrition is complex and heterogeneous in nature, ranging from larger categories of ‘Food and Nutrients’ to ‘Government and Regulation’, yet within each broad category, many subcategories are shared.

Beyond biochemical investigations, nutrition is distinct in its translation from research to practice as food is personally and culturally rich. While the first and foremost purpose of food for humans is to fulfill the biological need for energy and nutrients, the nature of food intake has biological and cultural cues. Food choices and preparation, number of meals per day, time of eating, method of eating (23), religious observation and personal food beliefs (24–26) are just a few examples of how a culture or custom may guide nutritional intake. Access and management of resources can also impact food selection and consumption, as individuals may have limited access to nutritious and/or preferred food items based on location and transportation needs (27). Individuals with limited monetary resources are also forced to make decisions between food and other necessities such as housing, which can further impact health and safety (28). The sociological implications of food greatly impact an individual or population’s nutritional intake and quality of life in ways that are not captured from a purely biochemical point of view. This complex nature of food and nutrition creates a highly variable notion as to what ideal nutrition is while also showcasing how integral food and nutrition are to human daily life and biological function. Due to the deep complexity of nutrition, discussion of health outcomes involving nutrition is arguably incomplete without the inclusion of sociological information. The broad biological, behavioral and resource-driven scope of nutrition and nutrition research is illustrated in Figure 1, depicting how broad categories of nutrition are all interconnected by subcategories. Due to the interrelated nature of nutrition as a whole, nutrition data and research must also be managed in a unified fashion.

Because nutrition is interdisciplinary and heterogeneous, it is an emergent area for the application of informatics, particularly due to the recent increases in data production through various -omics based nutrition research. The desire to utilize nutri-informatics approaches to interpret nutrition data can be guided by the successful use of integrated informatics approaches in other biomedical fields, such as genomics, transcriptomics and metabolomics in combination with more traditional epidemiological and statistical approaches. Currently, nutrition data range widely including for example survey data, clinical data, basic science mechanism data, observational data and -omics data.

## Nutri-informatics progress toward improved disease management and precision health

While nutri-informatics may appear to be a new trend, the application of nutri-informatics using advanced statistics has been pursued in nutrition research for some time within large-scale investigations of dietary intake via surveys. Surveys such as What We Eat In America (WWEIA), a subset of the National Health and Nutrition Examination Survey (NHANES), are collected biannually from Americans in an effort to depict nutritional intake and correlate it with biological samples and clinical measures collected via NHANES (29). Since the initiation of WWEIA in the 2003–2004 survey period, investigators have capitalized on access to these nutrition data sets for research. Research projects that utilize data from WWEIA and NHANES range widely in focus, such as investigations into cost and energy intake associated with dairy replacement in individuals who do not consume dairy products (30), and the prevalence of probable undiagnosed celiac disease and potential reduction in femur bone mineral density (31). The Nurses’ Health Study (NHS), now in its third iteration, similarly collects longitudinal dietary information within their large cohorts via semiquantitative food frequency questionnaires (FFQs) (32). Since its initiation, NHS dietary information correlated with biological specimens and clinical outcomes in participants have been used to evaluate potential biomarkers for nutrition, such as being the first investigation to measure intake of selenium via toenail samples (32). Furthermore, NHS has also informed dietary guidelines, such as the recommendation to reduce or eliminate trans-fatty acids from the diet to reduce coronary heart disease (32) and highlighting the correlation between eating patterns such as Dietary Approaches to Stop Hypertension and prevention of colorectal cancer in men (33). Nutrition surveys such as WWEIA and NHS have enabled epidemiological nutrition evaluations with advanced statistics and correlation with clinical and biospecimen data to support improved public health recommendations. While survey-based investigations continue to produce nutrition data to support epidemiological research, approaches to gathering related data have expanded to new data types and a requirement for new methods to incorporate data sources for analysis and inference.

Nutri-informatics initiatives often fall into the category of clinical nutrition, with researchers utilizing informatics to integrate data from electronic health records (EHRs), patient surveys, wearable devices, mobile applications and other tools to facilitate inference of optimal health recommendations. The Academy of Nutrition and Dietetics, the largest professional group of registered dietitian nutritionists in the USA, is striving to participate in the development of standards and processes using nutri-informatics to facilitate optimal nutrition care (34). This has included support for transitions to EHRs as well as standardization of the electronic Nutrition Care Process and Terminology (eNCPT), a systematic terminology that describes nutrition patient care through Assessment, Diagnosis, Intervention, Monitoring and Evaluation (34, 35). eNCPT also integrates with the Systematized Nomenclature of Medicine—Clinical Terms and Logical Observation Identifiers, Names and Codes, two commonly used medical terminologies (34). Implementation of eNCPT in care settings has documented improved efficiency and increased nutrition-related diagnoses in hemodialysis patients compared to manual paper-based systems, supporting greater effectiveness in patient outcomes (36). Another approach includes malnutrition identification within a hospital setting (37). Malnutrition is an extreme risk for hospitalized patients, exacerbating chronic and acute health conditions such as reduced immune function and impaired wound healing and potentially increasing morbidity and mortality rates (37). Software-based screening tools that standardize malnutrition assessments have improved the consistency and efficiency with which malnutrition is diagnosed, expediting the nutrition care response for patients (37), and also supported the malnutrition knowledge, attitudes and practices of staff (38).

Nutri-informatics has also been applied in the context of personalized nutrition, i.e. an individual’s personal diet and how it translates to health and well-being (39). As both health and disease are highly variable based on genetics, lifestyle, environmental exposures and many other factors, researchers are focusing on inclusive approaches to develop data-driven predictive methods for anticipating an individual’s response to food (39). An investigation into personalized nutrition for glycemic control by Zeevi *et al*. utilized machine learning techniques tracking anthropometrics, dietary intake, individual microbiome and glycemic status to develop a predictive model for postprandial glucose response (PPGR) (14). These findings displayed the extreme variability in PPGR seen across individual participants, denoting the importance of personalized nutrition approaches in comparison to broad population-based recommendations (14).

Understanding food intake is a critical part of evaluating both population and personalized nutrition, and informatics approaches have also been used to track food purchasing and food intake and correlate it with nutrition information. One investigation examined whether grocery store purchases could be associated with specific nutrition information from a U.S. Department of Agriculture (USDA) database. The study found that most food products could be accurately mapped to nutritional composition (40). While this investigation faced barriers in mapping inconsistencies across food categories that have highly variant nutritional content, 70% of food items were mappable to the USDA nutrient database and 100% of items were mappable to USDA standard food groups. The investigators described a feasible approach for interpreting nutritional intake of grocery store purchases and expressed that greater interoperability between nutrition information and food labeling and production systems as well as healthcare would support translation of this type of research (40).

Utilizing electronic food diaries and phone applications has become a popular approach to documenting and analyzing dietary intake. Generally, electronic food diaries offer a wide range of functionality and the ability to store and share data across users, with aggregation and summarization of food intake and inferences on health outcomes offering users the most benefit (41). Some versions of electronic food diaries within mobile device applications may include users sharing pictures of food they have consumed. With these types of applications, approximately a quarter of them provide either professional or crowdsourced feedback from other users (42). Notably, most photo-based apps are conducted with little to no application of evidence-based methods for self-regulation and behavior change, which may impact user health behaviors and outcomes (42). Beyond crowdsourced and professional responses to photos, one group of researchers developed an image recognition algorithm to recognize and analyze nutrition content from a photo of food (43). This dietary tracking system, DietLens, utilizes deep-based food recognition technologies to classify the image and applies neural networks for image-level food categorization (43). Thus far, this technology has been able to categorize food images from the research testing laboratory with between 75% and 99% accuracy, although difficulties were seen with mixed dishes that contain a large variation in ingredient composition (43). When compared to other electronic food diary apps, DietLens displayed greater accuracy and required less time to log nutritional intake, indicating photo recognition–based applications may be a useful tool for personal dietary intake tracking (43).

Plant and animal nutrition can also be factors in human nutrition, and informatics approaches have been used to evaluate crop breeding and quality, as well as animal genomics. High-throughput approaches for crop genotyping and phenotyping are becoming popular approaches to understanding plant genetics and breeding. Databases such as Germinate 3 have been developed in an effort to store, visualize and analyze data from crops such as potatoes, barley and wheat (44). This publicly available database allows for queries of the data to optimize knowledge gains for the user to facilitate desired crop cultivation.

For farm animals, researchers have displayed a wide array of interests that range from pathology and physiology studies to DNA isolation for meat quality assurance (45). Nutri-informatics in this space has been particularly valuable for animal breeding, with sequencing of pig genomes for example allowing not only for investigation into clinical studies using this model organisms (46), but also for breeders looking to produce pigs that are resistant to infectious disease (47). Similar approaches have been seen in chickens, fish and other organisms that serve as meaningful models for human health as well as common dietary components. Of note, genomic standards have become a key factor in facilitating these kinds of discoveries, including Minimum Information about any (x) Sequence (MIxS) (e.g. genome sequences) and related minimum information standard checklists that serve as modular and extensive standards for reporting sequence data in public sequence repositories (48).

Overall, current progress in nutri-informatics research is promising and has given rise to novel findings and methodologies that can likely be utilized in future research endeavors. However, many barriers are still limiting the ability to bring nutri-informatics to the forefront of precision medicine and personalized health.

## Current nutri-informatics challenges

Many clinical settings have focused on transitioning to electronic resources for nutritional data documentation and storage, allowing for widely accessible albeit static clinical measurements. However, the multitude of methods for capturing nutritional information within EHRs and the lack of standardization across them limits their research use (39). Furthermore, nutrition data are often sparse within EHRs, limiting the capacity to evaluate potential nutritional impacts on health outcomes (39). As such, clinical nutri-informatics investigations more often focus on specific health outcomes such as specific disease states and clinical biomarkers as opposed to larger, more integrative studies that incorporate a wider array of data types and outcomes. A good example is the implementation of malnutrition screening assessments, which although have been important for identifying and managing malnutrition, a validated Malnutrition Screening Tool asks only two questions, ‘Have you lost weight recently without trying? If yes, how much weight have you lost?’ and ‘Have you been eating poorly because of a decreased appetite?’ (49). With just two questions asked via patient survey, no dietary intake or other information is acquired. This allows for the clinician to identify malnutrition at a gross level but provides little insight into any specific dietary factors. Very few attempts have been made to try and utilize nutrition and clinical survey data, -omics data and other heterogeneous data types in coordination together in research.

Furthermore, major challenges exist for personalized nutrition endeavors, including experimental designs being unable to track the complex physiological response to nutrient exposures (nutrients and potential contaminants, food additives or toxins), incomplete understanding and establishment of metabolic biomarkers, and inconsistent documentation language or incorrect reporting of health exposures and outcomes (39). Concerns with self-report survey approaches also occur, as participants may inaccurately depict their nutritional intake, challenging research findings. In one investigation, average 24-h dietary recalls underestimated dietary sodium intake, when compared to estimated consumption calculated from 24-h urine sodium content (50). Concerns for racial disparities utilizing FFQs also arise. One investigation identified significantly greater correlation between 24-h dietary recall and FFQ for white women compared to black women, challenging the ability to decipher eating habits and make dietary recommendations given the racial disparities not captured within nutrition surveys (51).

Nutrition research generally faces challenges, with a variety of research methodologies available and each with trade-offs of benefits and challenges. While single dietary element investigations are optimal for evaluating nutrition biomarkers, investigations that single out a particular dietary component can be challenging to complete in a controlled manner in humans and may also lead to broad interpretations regarding the systemic effects of the food or nutrient in health. On the opposing side, many investigations evaluate diet in its entirety, which limits the capacity for evaluation of specific cellular and molecular interactions or signaling (39). For lack of a ‘perfect’ methodological design, many nutrition investigations are conducted on similar concepts producing a wide range of data and data types that are not compatible enough for larger-scale insights, limiting opportunities for translational research and further hypothesis development.

**Figure 2.**
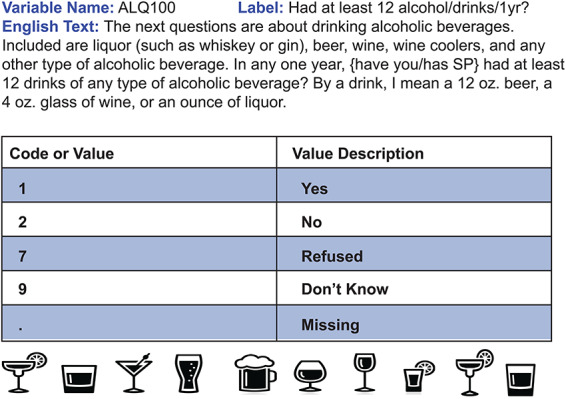
Sample CDE. This question is an example of a CDE from the NHANES 1999–2000 questionnaire. In this instance, survey participants were inquired about alcohol consumption throughout the year and their responses were standardized using the corresponding code/value pair. Usage of this CDE in a separate survey, such as a future year of NHANES, will allow data from both surveys to be directly comparable.

Biomedical data as a whole are represented using a wide array of terminologies for similar or identical concepts, leading to challenges for data aggregation and management even with smaller data sets (52). The National Institute of Health (NIH) has created common data elements (CDEs), which are standardized key terms or concepts, established so that they may be used in clinical research or in studies, to enhance data quality and so that the data can be used across sites and over time (https://cde.nlm.nih.gov/). CDEs are designed to support data collection and analysis in a consistent fashion (52) for a variety of data types such as from surveys, clinical data and laboratory findings. CDEs are intended to be reused within and across projects, meaning two different assessments can ask the same question using the same CDE format and the data produced from the question will be compatible between surveys or studies. An example of a CDE used in the NHANES 1999–2000 questionnaire is depicted in Figure 2 (https://wwwn.cdc.gov/Nchs/Nhanes/1999-2000/ALQ.htm).

Utilizing CDEs, nutrition research and data could be approached with standardization and interoperability in mind, similar to the structure CDEs have provided to projects within the fields of cancer research (52) and stroke clinical and epidemiological research (53). Currently, locating and identifying suitable CDEs can still pose some challenges. Searching through available CDEs using a general term like ‘nutrition’ will result in hundreds of CDEs related to food consumption, which can be read through to identify the ideal option. Unfortunately, underrepresented areas in research are similarly underrepresented in CDEs such as questions regarding food security or cultural dietary intake. While growth is still needed in this area, CDEs are a step in the right direction.

CDEs and other scientific records like data sets can be managed via unique persistent identifiers (PIDs), which facilitate data sharing, reuse and attribution (54). While CDEs and PIDs for data reuse and sharing are commonly used in other areas of biomedical sciences, CDEs are not in widespread use in nutrition investigations. Furthermore, researchers have seldom discussed nutri-informatics research from the perspective of data reuse to maximize understanding and comparability of findings (55).

In general, the lack of standardization and the technical issues present in nutri-informatics research causes great concerns within the community (Table 1). To alleviate the technical concerns within the nutri-informatics domain, a systematic approach with community-wide support and adoption is necessary.

**Table 1.** Technical challenges and needs for nutri-informatics

Needs	Rational	Example of application
Consistent terminologies	Inconsistent terminologies can cause difficulty for researchers and readers of the literature to understand the exact entity and definition being utilized leading to misinterpretations and poor reuse of data and/or outcomes.	Development of thesauri and other terminologies intended to provide consistent language for a domain of interest. An example of this is standardized language documenting the appropriate terminology for nutrients in a mammalian diet.
Use of persistent, stable identifiers	Persistent, stable identifiers ensure a term, data set or other element is coordinated with the correct metadata in a fashion that is not at risk of deletion or reassignment regardless of time passing.	A researcher looking to investigate the impacts of zucchini consumption on health can evaluate previous investigations on ‘zucchini’ through its persistent, stable identifier. They can also track information on ‘courgette’ as that term is also connected to the same identifier.
Coordinated standards across domains	Standards that span across all realms of biology are non-existent and for domains that do utilize standards, lack of alignment with other domains can limit the interoperability and coordinated usage of data.	Creation and alignment of standards for closely related fields such as genomics and toxicology would make documentation and comprehension of data easier for researchers working in both fields. This could span across fields of biology, offering the same benefits to a wide audience of researchers.
Clear documentation	While standards may exist for a variety of disciplines, ensuring they are well documented in a fashion that community members can use them is essential for their actual implementation.	GitHub is a common platform for documentation and community discourse for bioinformatics research communities. Terminologies, instructions for use and areas to suggest improvements are available and open for community member input.
Open access and use	To support the use of terminologies, data and other resources on the basis of scientific merit rather than restrictions of licenses, open access is highly preferred to allow referencing at any time by anyone.	Freely available and open-source terminologies would allow for researchers globally to discuss nutrition concepts (e.g. diet patterns, nutrients and dietary supplements) in a consistent fashion regardless of the researcher’s funding and access to licensed content.

## Biomedical ontologies can support standardization and integration of nutrition data

One approach to develop structure and standardization needed in nutri-informatics (Table 1) is the use of ontologies. Ontologies are classifications of terms focused on specific areas of knowledge or domains that include logically defined relationships between the terms (56). Ontologies are intended to be developed with the goal of consistent terminologies, coordination of data elements and standardized development to support interoperability and data reuse. PIDs are frequently used in ontologies and identifiers from other sources (e.g. nomenclature from the International Union of Pure and Applied Chemistry (IUPAC)) to maintain coordination across information sources.

Ontologies offer not only human readable definitions of terms, but also computer readable definitions in the form of logical definitions or axioms The logical definitions in ontologies leverage the computable relationships documented in efforts to inform hierarchies, similar terms or connections represented across ontologies. These relationships are also standardized and housed within ontologies like the Relation Ontology (RO), which documents and defines relationships suitable to depict relationships for ontologies (57). This allows for reasoning across the data and increased computability (56, 58–60). While ontologies are created specifically for a domain or subdomain, many ontologies are co-developed to be interoperable and compatible with one another making it easier to exhibit relationships between terms in different ontologies (56). Ontologies have been applied extensively in areas such as genomics and phenomics, which has allowed for increased connections between patient genotypes and clinical phenotypes, facilitating individualized medicine and rare disease identification (59–61).

Prominent ontologies frequently used in biomedical research include the Gene Ontology (GO) describing gene functions and biological processes (62) and the Systematized Nomenclature of Medicine—Clinical Terms (SNOMED-CT) that is a clinical terminology for medical conditions and symptoms (63). While there are a variety of analyses and applications for utilizing ontologies, two common approaches are similarity comparisons and enrichment analyses. An example of a semantic similarity comparison is the use of non-exact phenotype profile matching. Using patient profiles encoded with phenotype terms from the Human Phenotype Ontology (HPO), multiple profiles can be compared to identify similar and unique phenotypes between them. The application of semantic similarity algorithms over ontology-encoded clinical phenotype data for ‘fuzzy’ phenotype matching has supported diagnosis of rare disease patients (64, 65).

Enrichment analyses are also common approaches to utilizing ontologies. For example, an investigation looking to evaluate changes in vitamin D and serotonin gene expression for individuals with irritable bowel syndrome (IBS) assessed gene transcripts of tissue biopsy samples from IBS+ and IBS− populations (66). After identifying genetic features of interest via differential expression, enrichment utilizing GO highlighted the associated pathways and functions of the differentially expressed genes (66). In this instance, investigators identified the most prevalent enrichment within the serotonergic pathway, which paired with real-time PCRs may indicate that IBS patient-derived RNA has lower tryptophan hydroxylase-1 expression, which is a rate-limiting step in serotonin synthesis (66).

Organizations such as the Open Biomedical Ontology (OBO) Foundry have been particularly influential in the development of ontologies, providing a variety of community principles for best practices such as versioning, strong documentation, open access and common formats (67). These kinds of efforts have allowed for the use of ontologies in scientific research to grow substantially over the past few decades. However, their use within the discipline of nutrition has lagged regardless of researchers exhibiting a need for nutrition data standardization through the application of ontologies (55). Integration of nutrition into biomedical ontologies holds the potential to identify hundreds of nutrition–disease, nutrition–phenotype and nutrition–genotype relationships.

The complexity and coverage differ greatly across nutrition subdisciplines but many nutrition-related knowledge resources do exist including some which can be leveraged to better understand nutrition and human health in a computable manner. In Tables 2 and 3, a review of prominent existing resources is provided, including food and nutrition focused ontologies and related biomedical knowledge resources.

**Table 2.** A listing of prominent nutri-informatics ontologies

Chemical Entities of Biological Interest (ChEBI) (68) https://www.ebi.ac.uk/chebi/	Description: A dictionary of molecular entities focused on ‘small’ chemical compounds. Includes chemical dietary metabolites and nutrients.
	Example Term Name: L-ascorbic acid
	Example Term ID: http://purl.obolibrary.org/obo/CHEBI_29073
	Example Term Definition: The L-enantiomer of ascorbic acid and conjugate acid of L-ascorbate.
	Example Term Synonyms: (5R)-5-[(1S)-1,2-dihydroxyethyl]-3,4-dihydroxyfuran-2(5H)-one L-threo-hex-2-enono-1,4-lactone
Ontology of Nutritional Studies (ONS) (69) https://github.com/enpadasi/Ontology-for-Nutritional-Studies	Description: A systematic ontology framework for nutritional studies. Includes nutrition study design and diets.
	Example Term Name: Intervention Diet
	Example Term ID: http://www.enpadasi.eu/ontology/release/v1/ons/ONS_0000081
	Example Term Definition: The diet administered during an intervention study. It usually comprises the adoption of a certain nutritional intervention, intended as the prescription of consuming or not consuming certain food, and follows a precise study design. Intervention studies usually compare at least two subgroups of a population, one control group receiving a null nutritional intervention, and one or more test groups receiving the intervention.
Gene Ontology (GO) (62) http://geneontology.org/	Description: A computational model of functions of genes including metabolism related biological processes.
	Example Term Name: Diacylglycerol metabolic process
	Example Term ID: http://purl.obolibrary.org/obo/GO_0046339
	Example Term Definition: The chemical reactions and pathways involving diacylglycerol, a glyceride in which any two of the R groups (positions not specified) are acyl groups while the remaining R group can be either H or an alkyl group.
	Example Term Synonyms: diacylglycerol metabolism diglyceride metabolism
Food Biomarker Ontology (FOBI) (70) https://github.com/pcastellanoescuder/FoodBiomarkerOntology	Description: A joint ontology including Food Ontology and Biomarker Ontology including food intake biomarkers.
	Example Term Name: 3,4-dihydroxyphenylacetic acid
	Example Term ID: http://purl.obolibrary.org/obo/FOBI_030377
Medical Actions Ontology (MAxO) https://github.com/monarch-initiative/MAxO	Description: A structured vocabulary for medical procedures, interventions, therapies and treatment including medical nutrition therapy.
	Example Term Name: Dietary intervention
	Example Term ID: http://purl.obolibrary.org/obo/MAXO_0000088
	Example Term Definition: Any alteration or treatment in an individual’s diet with a planned goal, usually designed to improve the individual’s overall health.
	Example Term Synonyms: Behavioral nutritional intervention diet
Environmental Conditions, Treatments and Exposures Ontology (ECTO) https://github.com/EnvironmentOntology/environmental-exposure-ontology	Description: A structured vocabulary for environmental exposures and stimuli including food, nutrient and diet exposures.
	Example Term Name: Vitamin D exposure
	Example Term ID: http://purl.obolibrary.org/obo/ECTO_9000133
	Example Term Definition: An exposure to vitamin D.
	Example Term Synonyms: Exposure to vitamin D
Human Phenotype Ontology (HPO) (71) https://hpo.jax.org/app/	Description: A standardized vocabulary of phenotypic abnormalities encountered in human disease including nutrition-related phenotypes.
	Example Term Name: Abnormality of amino acid metabolism
	Example Term ID: http://purl.obolibrary.org/obo/HP_0004337
	Example Term Definition: Abnormality of an amino acid metabolic process.
	Example Term Synonyms: Amino acid levels abnormal
Mondo Disease Ontology (Mondo) https://mondo.monarchinitiative.org/	Description: A harmonization of disease definitions including nutrition-related diseases.
	Example Term Name: Protein–energy malnutrition
	Example Term ID: http://purl.obolibrary.org/obo/MONDO_0001371
	Example Term Definition: A nutritional deficit that is caused by inadequate protein or calorie intake.
	Example Term Synonyms: Protein–energy malnutrition
Food Ontology (FoodOn) (72) https://foodon.org/	Description: An ontology focused on categorization and processing of food including terminology for food, food components and food management.
	Example Term Name: Macaroni and cheese
	Example Term ID: http://purl.obolibrary.org/obo/FOODON_00002960
	Example Term Definition: Macaroni and cheese is a dish of English origin, consisting of cooked macaroni pasta and a cheese sauce, most commonly cheddar. It can also incorporate other ingredients, such as breadcrumbs, meat and vegetables.
	Example Term Synonyms: mac n cheese macaroni cheese mac and cheese
Crop Dietary Nutrition Ontology (CDNO) (73) https://github.com/Southern-Cross-Plant-Science/cdno	Description: An ontology aimed to assist management and navigation of dietary nutritional components derived from crops. An initial version of this ontology is anticipated to be available in 2021.
Neuro Behavioral Ontology (NBO) (74) https://github.com/obo-behavior/behavior-ontology	Description: An ontology of human and animal behaviors and behavioral phenotypes including motivation and behaviors regarding food and beverage consumption.
	Example Term Name: Polyphagia
	Example Term ID: http://purl.obolibrary.org/obo/NBO_0000546
	Example Term Definition: A pathological eating behavior characterized by an abnormally large intake of food by mouth, usually due to excessive hunger that is relatively prolonged.
	Example Term Synonyms: Hyperphagia
Sustainable Development Goals Interface Ontology (SDGIO) (75) https://github.com/SDG-InterfaceOntology/sdgio	Description: This ontology serves to clarify the nature of an interlinkage between entities references by the Sustainable Development Goals and their targets and indicators.
	Example Term Name: Water-borne disease
	Example Term ID: SDGIO:00010019
	Example Term Definition: A disease that is transmitted via a medium composed of water.
Systematized Nomenclature of Medicine—Clinical Terms (SNOMED CT) (63) http://www.snomed.org/snomed-ct/why-snomed-ct	Description: Comprehensive clinical terminology including nutrition-related clinical terminology.
	Example Term Name: Vitamin D overdose (disorder)
	Example Term ID: SCTID: 296953002
	Example Child classes: Accidental vitamin D overdose (disorder) Intentional vitamin D overdose (disorder) Vitamin D overdose of undetermined intent (disorder)
International Classifications of Disease (ICD) https://icd.who.int/en	Description: The global standard for diagnostic health information including coding and billing terminology for nutrition diagnostics.
	Example Term: Wernicke’s encephalopathy
	Example ICD-10-CM Code E51.2
	Example Term Definition: Wernicke’s encephalopathy (or Wernicke’s disease) refers to the presence of neurological symptoms caused by biochemical lesions of the central nervous system after exhaustion of vitamin-B reserves, in particular thiamine (vitamin B1). The condition is part of a larger group of diseases related to thiamine insufficiency, including beriberi in all its forms and Korsakoff syndrome. When Wernicke’s encephalopathy occurs simultaneously with Korsakoff syndrome it is known as Wernicke–Korsakoff syndrome.
	Example Specialty: Endocrinology
Medical Subject Headings (MeSH) (76) https://www.nlm.nih.gov/mesh/meshhome.html	Description: A vocabulary of biomedical and health-related information including nutrients and food components.
	Example Term Name: Niacin
	Example Term ID: D009525
	Example Term Definition: A water-soluble vitamin of the B complex occurring in various animal and plant tissues. It is required by the body for the formation of coenzymes NAD and NADP. It has PELLAGRA-curative, vasodilating and antilipemic properties.
	Example Entry names: 3-Pyridinecarboxylic Acid Niacin Aluminum Salt Niacin Ammonium Salt Niacin Calcium Salt

**Table 3.** A listing of prominent nutri-informatics databases and resources

Comparative Toxicogenomics Database (77) http://ctdbase.org/	Description: A publicly available data set of environmental exposure impacts on human health including representation of food chemical and metabolite interactions and nutrition-related disease, phenotype and gene associations.
	Example Term Name: Folic Acid
	Example Term Definition: A member of the vitamin B family that stimulates the hematopoietic system. It is present in the liver and kidney and is found in mushrooms, spinach, yeast, green leaves and grasses (POACEAE). Folic acid is used in the treatment and prevention of folate deficiencies and megaloblastic anemia.
	Example Top 3 Gene Interactions: SLC19A1, TNF, AGT
	Example Term Synonyms: B9, Vitamin | Folacin | Folate | Folic Acid, Calcium Salt (1:1) | Folic Acid, (D)-Isomer | Folic Acid, (DL)-Isomer | Folic Acid, Monopotassium Salt | Folic Acid, Monosodium Salt | Folic Acid, Potassium Salt | Folic Acid, Sodium Salt | Folvite | Pteroylglutamic Acid | Vitamin B9 | Vitamin M
NutriGenomeDB (78) http://nutrigenomedb.org/	Description: A nutrigenomics exploratory and analytical platform including nutrigenomics gene expression data modules.
	Primary Features: Gene Expression Browser—Includes gene expression database search tools and expression heat map generation tool. Phenotype-Centered Analysis—Evaluates a list of differentially expressed genes and aggregates similar experiments based on gene expression profiles characterizing specific phenotypes.
The Monarch Initiative (79) https://monarchinitiative.org/	Description: An integrative data and analytic platform connecting data across species including nutrition-related phenotypes, genotypes and diseases.
	Example Term Name: Rickets (disease)
	Example Term ID: http://purl.obolibrary.org/obo/MONDO_0005520
	Example Term Definition: Bone softening and weakening usually caused by deficiency or impaired metabolism of vitamin D. Deficiency of calcium, magnesium or phosphorus may also cause rickets. It predominantly affects children who suffer from severe malnutrition. It manifests with bone pain, fractures, muscle weakness and skeletal deformities.
	Example Exact Synonyms: Vitamin D-dependent rickets, rachitis, vitamin D hydroxylation-deficient rickets, rickets. Example Related Synonyms: Active rickets, vitamin-D deficiency rickets, hypovitaminosis D, nutritional rickets, vitamin D deficiency disease
	Example Related Phenotypes: Short stature, tibial bowing, recurrent fractures
	Example Genes (causal): PHEX, FGF23, SLC34A3
USDA FoodData Central (FDC) https://fdc.nal.usda.gov/index.html	Description: An integrated data system with nutrient profile data and links to related research including nutrient profiles for common foods and beverages.
	Example Term Name: Beans, black turtle, mature seeds, canned
	Example FDC ID: 175 188
	Example Food Category: Legumes and Legume Products
	Available data includes: Macronutrient content Micronutrient content Average weight/volume
USDA National Health and Nutrition Examination Survey (NHANES) and What We Eat in America (WWEIA) (29) data sets https://www.cdc.gov/nchs/nhanes/index.htm	Description: Data sets of compiled health and nutrition status information for adults and children.
	NHANES available data: Biological samples of serum, plasma, urine and DNA Demographic, Dietary, Examination, Laboratory and Questionnaire health data WWEIA available data: 24-hour dietary recalls (2) Type and amount of food and beverage consumed Time, location and name of eating occasion Water consumption type and source Use of table salt Special diets and usual daily intake Calculated daily total intake of energy and >60 nutrients/food components
FoodEx2 (80) https://www.efsa.europa.eu/en/data/data-standardisation	Description: A standardization effort from the European Food Safety Authority (EFSA) aimed to cover the needs to describe foods in data collections across various food safety domains.
	Example Base Term: Tomato-containing cooked sauce
	Example facet descriptors: Ingredients: basil, garlic Processing: jarring, pasteurization Packaging format: jar Packaging material: glass
European Food Information Resource (Eurofir) (81) https://www.eurofir.org/	Description: A membership-based international non-profit aiming to develop, publish and exploit food composition information, and promote international cooperation and harmonization of standards to improve data quality, storage and access. Example Term Name: Aspartame
	Example Term Code: ASPM
	Example Term Additional Information: <INFOODS>ASPM Artificial sweetener EuroFIR priority: 7

Nutri-informatics resources are largely focused on clinical nutrition, foods and nutrients. Terminological resources such as the Mondo Disease Ontology, the HPO, SNOMED-CT and International Classification of Diseases (ICD) have nutrition-related diseases and phenotypes. The Monarch Initiative and the Comparative Toxicogenomics Database (CTD) denote relationships between nutrients, disease, phenotypes and genes. Because nutrition can impact diseases or phenotypes that do not have an exclusively nutrition-based etiology, it is imperative that such relationships are discovered and included in these resources. Gene pathways for metabolism are represented in GO and nutrient-gene expression analysis platforms such as NutriDB have been introduced. Also, initial modeling of nutrient exposures in the Environmental Conditions, Treatments and Exposures Ontology (ECTO), dietary patterns and interventions in the Ontology for Nutritional Studies (ONS) and nutrient therapies in the Medical Action Ontology (MAxO) have been represented, alongside a few nutrition-related behaviors in the Neuro Behavior Ontology (NBO). There is also a need for representation of public health nutrition investigations, and resources such as the Ontology for Nutritional Epidemiology (ONE) (82) are promising. Such knowledge representation is still emergent and will continue to grow in the years to come and require further nutrition representation.

Food nutrients and processing have strong representation in the Food Ontology (FoodOn), the U.S. Department of Agriculture Food Data Central (USDA FDC), FoodEx2 and the European Food Information Resource (Eurofir). Macro- and micronutrients are well represented in the Chemical Entities of Biological Interest (ChEBI), Medical Subject Headings (MeSH) and CTD. Food-related biomarkers and metabolites that can be identified in biological samples are seen in ChEBI and the Food-Biomarker Ontology (FOBI). However, the large multitude of foods and beverages available for consumption as well as the wide array of agricultural and processing techniques used with consumable products requires much more extensive representation. Furthermore, nutrition biomarkers are a developing field and these resources will require continual revision.

Some representation is also seen within the realms of sustainable human development and food security through the use of the Sustainable Development Goals (SDGs) Interface Ontology (75), which serves as a linkage to describe entities and targets within the United Nations SDGs (83). Notably, mentions of sustainability for the environment as well as human development are still lacking within ontologies. Additionally, representation is still limited in areas such as nutrition sociology (e.g. food behaviors, beliefs, culture, norms and nutrition literacy), public health nutrition policy and nutrition education. Importantly, relationships available for use within ontologies are also somewhat limited in their ability to be used specifically when discussing nutrition. RO and other initiatives to standardize relationship definitions and usage have created a wide array of generalizable relationships that can be used in nutrition context, but with some creativity that may risk incorrect usage or improper interpretations from humans or machines. Further enrichment of nutrition-specific relationship terms (e.g. contains nutrient or contains food) may serve as meaningful ways to represent simple aspects of food and dietary content, as well as the interactions between nutrition, single organisms, populations and ecosystems.

**Figure 3.**
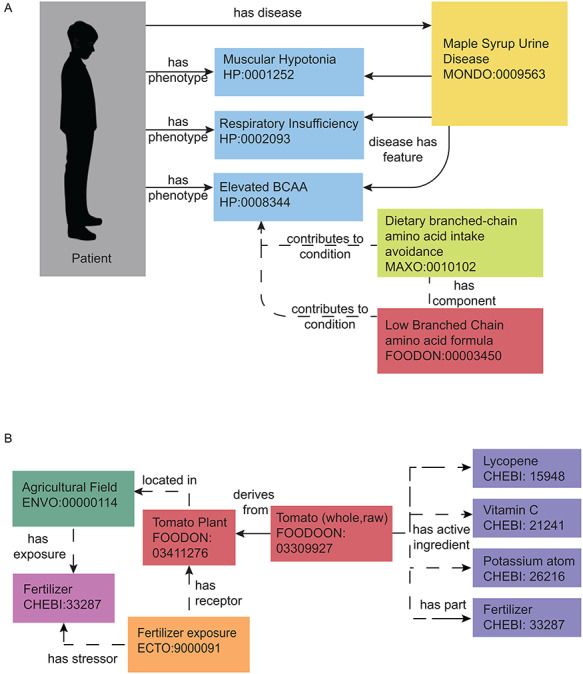
Representing nutrition using ontologies. Nutrition representation in current ontologies and databases is not yet sufficient to meet the needs of the nutri-informatics research community, yet some meaningful relationships can still be identified within the current landscape. Currently defined relationships can be seen with solid arrows and proposed modeling relationships can be seen in dashed arrows. (A) MSUD. This rare metabolic disease can be annotated with related phenotypes, nutritional recommendations and medical foods using interoperable biomedical ontology terms. These present and proposed relationships can be used to facilitate disease and therapeutic intervention identification with a set of patient phenotypes. (B) Farm to Fork with a Tomato. The process of growing a tomato can also be annotated by its exposures and nutrient content. Present and proposed relationships can connect fertilizer application at the field, to the food produced, to its nutrient and potential chemical content.

While nutrition and food representation in ontologies and databases will require substantial work from the ontology and data science and nutrition research communities to ensure adequate representation, there are still meaningful relationships represented in current resources. Figure 3 depicts meaningful relationships between food, agriculture, phenotypes and disease that can currently be represented using biomedical ontologies. In Figure 3A, for a patient presenting with a variety of phenotypes, logically the next questions might be what disease does this individual have and what are associated treatment options? Given the current structure of phenotype, disease and therapeutic terminologies currently within biomedical ontologies, the patient’s phenotypes can be connected with the rare Maple Syrup Urine Disease (MSUD). Additionally, given the common MSUD phenotype of ‘elevated branched chain amino acids (BCAA)’, a proposed associated therapeutic approach for that phenotype includes the use of a ‘Dietary branched-chain amino acid intake avoidance’ as well as the use of ‘Low branched chain amino acid formula’. Ontology content related to coordinated nutrition therapies and therapeutic foods are still largely in development, and further content in this area would be beneficial for similar modeling. In Figure 3B, if a patient were to present with a phenotype (e.g. kidney dysfunction from excess cadmium exposure) related to fertilizer exposure, one might be interested in how the individual is exposed to fertilizers in their current diet. Given a dietary questionnaire, the individual may report high intake of fresh tomatoes, which ontology terminologies may connect to the fertilizer exposure at the agricultural field growing the tomatoes, as well as the nutrients and chemicals present on the tomato prior to consumption. Modeling of this type of nutrient content and fertilizer exposure in agriculture is achievable using ontologies, but much is left to be developed to fully represent these types of connections.

## Opportunities for integrative nutri-informatics research

As organizations such as the NIH establish priorities for investigating nutrition exposures and precision nutrition for human health (84), the field of nutrition is in a meaningful position to launch investigations and continue growing our understanding of clinical dietary solutions for individuals and populations via nutri-informatics. In order to continue the integration of nutrition data into existing knowledge graphs and ontologies, comprehensive, standardized representation of all categories of nutrition from basic science to public policy is needed. This requires progressive integration of the many essential categories of nutrition seen in Figure 1, including foods and nutrients, clinical disease management through nutrition as well as those currently underrepresented categories such as sociological impacts on nutrition. In order to achieve this representation, there are multiple hurdles and opportunities that need to be addressed by the biomedical ontology and nutrition research communities:

1. Incomplete coverage of nutrition-related concepts in ontologies. Due to the widespread field of nutrition, representation for all subdisciplines of nutrition has yet to be achieved. Although efforts such as Food Ontology (FoodOn) (72) and Ontology of Nutritional Studies (ONS) (69) as well as others have made strides in representing foods and food components as well as nutrition intervention and epidemiological terminology, further areas such as nutrition sociology, nutrition policies and nutrition education are still limited.
2. Creation of new relationships and modeling of nutrition as a factor in disease and phenotype presentation, prevention and management is needed. While existing knowledge bases such as The Monarch Initiative (85) may address nutrition concepts like disease states from nutrient deficiencies, the focus is on other biomedical fields such as genomics and the impact on disease and phenotypes. It is likely that nutrition-related diseases and nutrition impacts on disease are underrepresented currently.
3. Limited compatibility across databases and knowledge resources containing nutrition-related information due to a lack of community development standards. Community standards for ontology and knowledge base development are not established for many nutrition resources, limiting their compatibility with other nutrition-focused and biomedically focused computable resources. Additionally, as more federal agencies and organizations with established vocabularies become interested in utilizing ontologies, more effort will be required to align and reuse existing resources to avoid duplication of work. A good example of such integration in ontologies is the Mondo Disease Ontology, which coordinates more than 17 different sources for an integrated representation of disease (86).
4. Poor communication and accountability regarding the Findability, Accessibility, Interoperability and Reusability (FAIR) principles of scientific data management and stewardship among nutrition researchers, nutrition journals and nutrition research funding agencies. FAIR have been designated as the foundational principles to guide data production and publication to support data transparency and maximize data outcomes (87). Due to the limited requirements or even recommendations for nutrition researchers to adhere to FAIR principles during their experimental process, scientific journals and research funding agencies limit the production and publication of optimal, computable data. This not only limits nutrition research findings but also hampers knowledge gains in related biomedical fields.

Given the lack of FAIR principles utilized and required for current nutrition research, and the need to integrate nutrition data and knowledge into existing knowledge bases, there is a need for standardized nutrition vocabulary, as well as best practices to encourage the curation of nutrition-related phenotypes, nutrition exposures, nutrition sociology and other nutrition subfields. This is especially true as investigations into environmental exposures, including nutrition, continue to be pursued in relation to the genome and gene expression (88).

## A call for improved nutrition representation and standards

In order to further our understanding of how nutrition and food impacts health, human behavior, culture and beyond, integrating nutrition terminology and relationships into ontologies and knowledge resources is essential. Increasing representation of nutrition in areas already being modeled in ontologies, such as foods, should be a focus, as well as areas yet to be explored in current resources. Areas including nutrition biomarkers, nutrition behavioral counseling, nutritional personal and cultural beliefs, and food processing can fall into this category.

The resources in Tables 2 and 3 have started striving toward representing nutrition in some capacity, but due to the vast nature of the field of nutrition, this representation is still incomplete for many topics as they have yet to be developed. Furthermore, some nutrition resources are not developed with compatibility in mind, further limiting interpretation and alignment of terminology across resources. These challenges in nutrition representation and compatibility across resources will require substantial, consistent work from the nutrition and ontology communities.

Working toward this goal of nutrition representation, a working group including curators from MAxO, ECTO, FoodOn, ONS, FOBI, the U.S. Department of Agriculture and other representatives are meeting regularly to discuss nutrition in the current ontology landscape. Thus far, this group has focused on how to represent diet, how to model an organism’s biological capacity to consume certain foods and the agricultural production related to foods. This working group functions via a GitHub page (https://github.com/FoodOntology/joint-food-ontology-wg) and is open to individuals or groups interested in participating.

**Figure 4.**
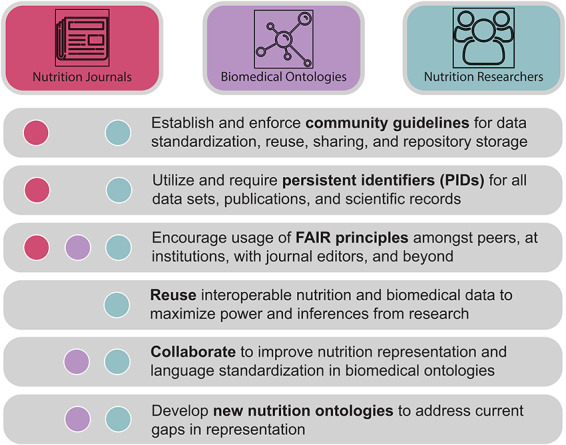
A call to action. Nutri-informatics stakeholders such as nutrition researchers, biomedical ontology developers and academic journal communities are needed to realize the connectivity and analyzability of nutrition data. Key tasks are described here, including actions to improve data interoperability, identifiability and collaboration between communities.

Beyond working groups such as this, further steps toward nutrition representation in this landscape are needed from nutrition researchers, academic nutrition journals and publishers, and biomedical ontology developers and curators, which are described in Figure 4.

Beyond standard development, continued collaboration and communication across the field of nutrition is necessary to achieve widespread usage and buy-in from stakeholders. Working groups, educational workshops and community decision-making practices would greatly benefit these efforts to facilitate greater usage of nutri-informatics resources and community standards. Furthermore, commitment and consensus from funding sources and academic journals to require community data management practices is an essential component to adoption of such practices by the community (89, 90). With greater understanding of the critical need for data and language standards in nutrition and the subsequent enforcement of those standards, nutrition researchers, journals and ontology curators can maximize the research outcomes in nutri-informatics and related biomedical fields, supporting data interoperability and reuse in biomedical sciences.

By representing nutrition semantics within biomedical ontologies, all currently represented biological fields can be correlated with nutrition and dietary exposures, including connections to diseases, phenotypes and genes. Beyond the clinical realm, representation of cultural food, agronomical practices, personal beliefs regarding diet, public health nutrition policies and the many other subdisciplines of nutrition all hold substantial potential for computable research in nutri-informatics.

With the utilization of biomedical ontologies and development of nutrition community standards for supporting FAIR principles, nutri-informatics research can progress to develop similar investigations to that of other fields. Nutrition data within ontologies may offer the ability to evaluate the impacts of dietary patterns, food combinations, pesticides and agricultural chemical exposures, cultural values and individual behavioral impacts on human health. While these nutri-informatics investigations may not be achievable with the current nutrition ontology resources, further development in this field will undoubtedly offer novel understandings of how nutrition impacts human life.

## Conclusion

Nutrition is a fundamental component to human and non-human animal life as an integral factor in the presentation of diseases, genes or phenotypes, as well as an influencing factor on behavior and culture. While modern nutrition research may be a ‘younger’ field of biology, it is far from insignificant and will require robust community standards in order to fully support FAIR practices, transparency and maximal knowledge gains from research. By utilizing standardized language and biomedical ontologies, nutrition data could be integrated into the larger scheme of biomedical knowledge bases, supporting interoperability and reuse. This integration work is already beginning with the initiation of new nutrition-focused ontologies and working groups. Through continued education and action from nutrition researchers and ontology developers to integrate nutrition research into biomedical ontologies, nutri-informatics investigations can grow to their full potential, supporting discovery from nutrition data beyond a single investigation and offering insights beyond the field of nutrition.
